# Systematic review: the effect of right hemicolectomy for cancer on postoperative bowel function

**DOI:** 10.1007/s00520-020-05519-5

**Published:** 2020-05-20

**Authors:** C. Hope, J. Reilly, J. Lund, HJN Andreyev

**Affiliations:** 1 Division of Medical Sciences and Graduate Entry Medicine, School of Medicine, University of Nottingham, Royal Derby Hospital, Uttoxeter Road, Derby, DE22 3DT UK; 2 Department of Hepatobiliary Surgery, Queens Medical Centre, Nottingham, NG7 2UH UK; 3 Department of Gastroenterology, Lincoln County Hospital, Greetwell Road, Lincoln, LN2 5QY UK; 4 School of Medicine, University of Nottingham, Nottingham, UK

**Keywords:** Colorectal cancer, Colorectal surgery, Bowel function, Right hemicolectomy, Gastrointestinal surgery, Adenocarcinoma

## Abstract

**Background:**

Right-sided cancer accounts for approximately 30% of bowel cancer in women and 22% in men. Colonic resection can cause changes in bowel function which affect daily activity. The aims are to assess the impact of right hemicolectomy for cancer on bowel function and to identify useful treatment modalities for managing bowel dysfunction after right hemicolectomy.

**Method:**

The review was conducted in line with PRISMA. Eligible studies evaluated the impact of right hemicolectomy on bowel function in those treated for colorectal neoplasia or assessed the effect of surgical technique or other intervention on bowel function after right hemicolectomy. Right hemicolectomy for inflammatory bowel disease or benign cases only were excluded. Articles were limited to studies on human subjects written in English published between January 2008 and December 2018.

**Results:**

The searches identified 7531 articles. Nine articles met the inclusion criteria, of which eight were cohort studies and one was a randomised trial. Loose stool, increased bowel frequency and/or nocturnal defaecation following right-sided colectomy occurs in approximately one in five patients. Some of these symptoms may improve spontaneously with time. Bile acid malabsorption and/or small bowel bacterial overgrowth may be the cause for chronic dysfunction. Some studies report that no or little difference in outcome between right-sided and rectal resections likely suggests poor function after right-sided resection.

**Conclusion:**

Right hemicolectomy can result in changes to bowel function. Patients should be counselled preoperatively, and follow-up should be designed to identify and effectively treat significantly altered bowel function.

## Introduction

Right-sided colon cancer accounts for approximately 30% of bowel cancer in women and 22% in men [1]. Earlier detection by screening programmes and advances in adjuvant treatments, combined with improved patient selection, means that the proportion of people surviving bowel cancer in the UK has more than doubled in the last 40 years [2]. Curative treatment for right-sided colonic cancer includes right hemicolectomy with or without adjuvant chemotherapy.

Colonic resection can cause changes in bowel function which affect quality of life. However, the focus of follow-up remains on detecting recurrent cancer, and it is clear that many patients struggle with very difficult bowel function after treatment [3], yet few patients are referred for specialist help [4].

The impact of rectal surgery on gastrointestinal function is well established [5–7], but there is much less emphasis on the functional impact of right-sided resections. Right hemicolectomy involves the removal of the ileocaecal valve and a variable length of terminal ileum, both of which play an important role in maintaining normal gastrointestinal function.

The aims of this systematic review are to assess the impact of right hemicolectomy for cancer on bowel function and to identify any treatment modalities that exist for treating bowel dysfunction after right hemicolectomy.

## Method

This systematic review was performed using Preferred Reporting Items for Systematic Reviews and Meta-Analyses (PRISMA) [8]. The review protocol is available on PROSPERO, registration number CRD4201811111.

### Eligibility criteria

Eligible studies evaluated the impact of right hemicolectomy on bowel function in those treated for colorectal neoplasia or assessed the effect of surgical technique or other intervention on bowel function after right hemicolectomy. Randomised controlled trials, case-control studies, cohort studies and meta-analyses were eligible. Studies on subjects under 18 years old, right hemicolectomy performed for only benign indications, case reports and studies that were not published as full articles were excluded. Right hemicolectomy for inflammatory bowel disease or benign cases only were excluded, as postoperative bowel dysfunction may be secondary to inflammatory bowel disease. Adjuvant chemotherapy was not an exclusion criterion. Articles were limited to studies on human subjects written in English published between January 2008 and December 2018, due to improvements in cancer care over the last 10 years.

### Information sources

The MEDLINE, Embase, Cochrane Library and PubMed databases were searched in conjunction with a clinical librarian for published articles. The reference lists of included articles were reviewed.

### Search

**Fig. 1:**
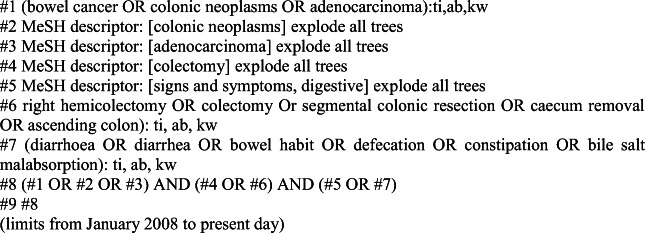
Cochrane Library search strategy

The search terms used were ‘bowel cancer’ OR ‘colonic neoplasms’ AND ‘right hemi-colectomy’ OR ‘colectomy’ OR ‘segmental colonic resection’. Figure 1 shows the search strategy for the Cochrane Database.

### Study selection

Titles, abstracts and full texts of articles were reviewed by two independent assessors (CH and JR), using Rayyan software [9]. In the event of a disagreement between the two reviewers, a third independent assessor opinion was sought (JL).

### Data collection process

The two reviewers independently extracted data from the included studies into an MS Excel spreadsheet. This is summarised in Table 1. Any disagreements regarding data extraction were resolved with discussion, and third reviewer opinion was sought if necessary. For each included article, study characteristics (methodology, setting, number of subjects, inclusion criteria) were recorded. The specific measurement tools employed and timing of administration along with type of resection performed were also recorded.

**Table 1:** Summary of all study characteristics

Author/year	Study	Measurement tool	*N* (right hemi)	Inclusion criteria	Type of resection	Study setting	Questionnaire timing	Findings
Palmisano, 2017	Retrospective cohort	Gastrointestinal Life Index EORTC QLQ-C30 EORTC QLQ-C29	225	2005–2014 Primary anastomosis only Cancer and ischaemic/inflammatory cases	Open and laparoscopic right hemicolectomy, extended right and ileocaecal resection	Italy	Pre-operation, 2, 6 weeks 3, 6 months	No significant impact on bowel function after ileocaecal valve removal Trend towards improvement in bowel symptoms over time
Theodoropoulos, 2013	Prospective observational	SF-36 EORTC QLQ-C30 Gastrointestinal Life Index EORTC QLQ-C29	85 (22)	> 18 years Elective cases with curative intent No major postoperative complications	Laparoscopic right hemicolectomy	Greece	1, 3, 6, 12 months	Health-related quality of life improves over the first year following all types of laparoscopic resection
Theodoropoulos, 2013	Prospective cohort	Gastrointestinal Life Index EORTC QLQ-C30 EORTC QLQ-C29	289 (79)	2007–2011 > 18 years Elective cases with curative intent	Laparoscopic right hemicolectomy	Greece	3,6, 12 months	Right hemicolectomy patients had less bowel dysfunction than other types of resection
Magdeburg, 2016	Retrospective cohort	SF-12 Faecal Incontinence Quality of Life scale	362 (85)	2005–2013 Cancer or diverticular disease Primary anastomosis	Open or laparoscopic right hemicolectomy	Germany	10–109 months	Right-sided more liquid stool than after left-sided resection
Brigic, 2017	Prospective cohort	EQ-5D Memorial Sloan-Kettering Cancer Centre Bowel Function questionnaire	261 (95)	Early group: recruited preoperatively Intermediate group: 2–4 years postoperatively Controls: healthy relatives Exclude: rectal tumours, previous pelvic radiation, previous abdominal surgery/stoma, prior anal incontinence	Open and laparoscopic right hemicolectomy	Not stated	6, 12 months 2 to 4 years	Worse frequency score for right-sided resection 2–4 years post-op
Ibanez, 2018	Double-blind randomised trial	Gastrointestinal Life Index > 3 liquid stools per day for >4 weeks	108	> 18 years Elective cases	Laparoscopic right hemicolectomy	Spain	1, 6, 12 months	No difference in type of anastomosis Higher diarrhoea rate in antiperistaltic
Ohigashi, 2011	Cohort	SF-36 EORTC QLQ-C30 Wexner Incontinence Score	124 (38)	2002–2006 Primary colorectal cancer		Japan	3 months	Right colectomy resulted in looser stool, increased nighttime defection than left-sided Probiotics significantly improved some aspects of questionnaire score
Thorsen, 2016	Prospective cohort	Diarrhoea Assessment Scale Gastrointestinal Life Index	98	< 75 years Elective cases only Cases: from ‘safe radical D3 right hemicolectomy for cancer through preoperative biphasic multi detector computed tomography’, 2012–2014 Controls: from hospital database, 2007–2014	Cases: right colectomy with D3 extended mesenterectomy Controls: right colectomy	Norway	14–34 months	Increased stool frequency in cases compared to controls Increased bowel frequency and urgency in both groups
Bertleson, 2018	Retrospective cohort	Bristol Stool Scale Number of bowel movements EORTC QLQ-C30	465	2008–2014 Elective right-sided resection for cancer Collected from retrospective database	Right hemicolectomy and extended right hemicolectomy vs right complete mesocolic excision	Denmark	2.11–5.53 years	Bowel dysfunction after right hemicolectomy is common (20%) 13% in conventional group had diarrhoea

*SF-36* 36-Item Short Form Survey, *SF-12* 12-Item Short Form Survey, *EORTC QLQ* European Organization for Research and Treatment of Cancer Quality of life questionnaire

### Risk of bias in individual studies

Methodology checklists for both cohort and case-control studies were reviewed, and relevant aspects from each were employed to critically appraise and grade the evidence of included studies. The quality of the included studies was assessed using the Cochrane Risk of Bias Tool [10] for randomised studies and STROBE criteria [11] for other study types. See Table 2.

**Table 2:** Studies meeting the STROBE statement recommendations

	Recommendation	Included in study
Title and abstract	(*a*) Indicate the study’s design with a commonly used term in the title or the abstract	2, 3, 5, 7
	(*b*) Provide in the abstract an informative and balanced summary of what was done and what was found	1–8
Background/rationale	Explain the scientific background and rationale for the investigation being reported	1–8
Objectives	State-specific objectives, including any prespecified hypotheses	1–8
Study design	Present key elements of study design early in the paper	1–8
Setting	Describe the setting, locations and relevant dates, including periods of recruitment, exposure, follow-up and data collection	1–8
Participants	(*a*) Give the eligibility criteria and the sources and methods of selection of participants. Describe methods of follow-up	1–8
	(*b*) For matched studies, give matching criteria and number of exposed and unexposed	5, 8
Variables	Clearly define all outcomes, exposures, predictors, potential confounders and effect modifiers. Give diagnostic criteria, if applicable	1–8
Data sources/measurement	For each variable of interest, give sources of data and details of methods of assessment (measurement). Describe comparability of assessment methods if there is more than one group	1–8
Bias	Describe any efforts to address potential sources of bias	
Study size	Explain how the study size was arrived at	8
Quantitative variables	Explain how quantitative variables were handled in the analyses. If applicable, describe which groupings were chosen and why	1–8
Statistical methods	(*a*) Describe all statistical methods, including those used to control for confounding	1–8
	(*b*) Describe any methods used to examine subgroups and interactions	1–8
	(*c*) Explain how missing data were addressed	
	(*d*) If applicable, explain how lost to follow-up was addressed	
	(*e*) Describe any sensitivity analyses	7
Participants	(a) Report numbers of individuals at each stage of study—e.g. numbers potentially eligible, examined for eligibility, confirmed eligible, included in the study, completing follow-up and analysed	1–8
	(b) Give reasons for non-participation at each stage	
	(c) Consider use of a flow diagram	2, 4, 7, 8
Descriptive data	(a) Give characteristics of study participants (e.g. demographic, clinical, social) and information on exposures and potential confounders	1–8
	(b) Indicate number of participants with missing data for each variable of interest	7, 8
	(c) Summarise follow-up time (e.g. average and total amount)	1–8
Outcome data	Report numbers of outcome events or summary measures over time	1–8
Main results	(*a*) Give unadjusted estimates and, if applicable, confounder-adjusted estimates and their precision (e.g. 95% confidence interval). Make clear which confounders were adjusted for and why they were included	5, 7, 8
	(*b*) Report category boundaries when continuous variables were categorised	1–8
	(*c*) If relevant, consider translating estimates of relative risk into absolute risk for a meaningful time period	
Other analyses	Report other analyses done—e.g. analyses of subgroups and interactions and sensitivity analyses	1–8
Key results	Summarise key results with reference to study objectives	1–8
Limitations	Discuss limitations of the study, taking into account sources of potential bias or imprecision. Discuss both direction and magnitude of any potential bias	1–8
Interpretation	Give a cautious overall interpretation of results considering objectives, limitations, multiplicity of analyses, results from similar studies and other relevant evidence	1–8
Generalisability	Discuss the generalisability (external validity) of the study results	
Funding	Give the source of funding and the role of the funders for the present study and, if applicable, for the original study on which the present article is based	5–7

*1* Palmisano, *2* Theodoropoulos (post-colectomy assessment), *3* Theodoropoulos (prospective evaluation), *4* Magdeburg, *5* Brigic, *6* Ohigashi, *7* Bertleson, *8* Thorsen

### Additional analyses

No meta-analyses or other statistical analyses were conducted due to the variation of study methodology and heterogeneity of results. Principally, the inclusion criteria and definition of right-sided resections varied widely between studies along with the different measures of bowel function, making direct comparison difficult.

## Results

**Fig. 2:**
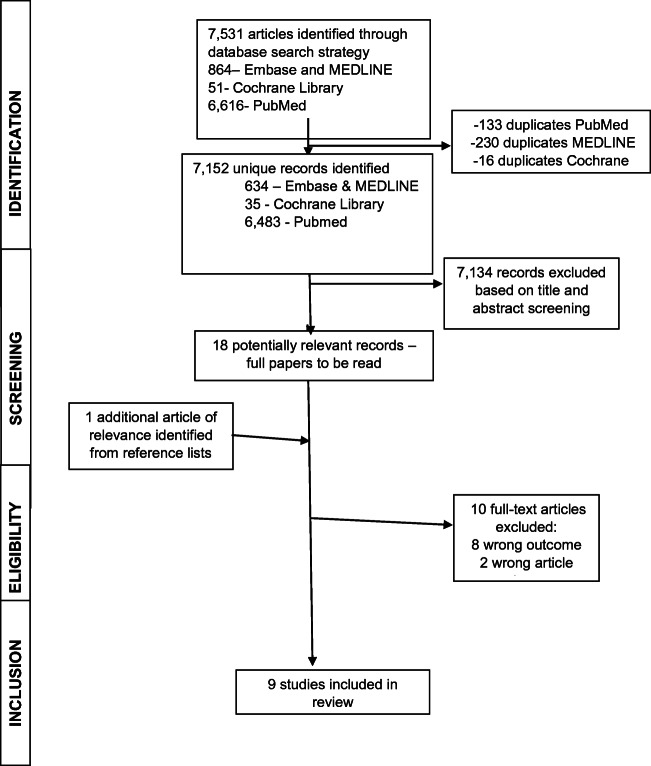
Flow diagram of study selection

The searches identified 7531 articles (Fig. 2). The main reason for exclusion on title and abstract screening was wrong population or wrong outcome. On full-text review, ten articles were excluded with reasons. Nine studies met the inclusion criteria and were evaluated and assessed for quality.

### Study characteristics

Of the nine articles, eight were cohort studies and one was a randomised trial (Table 1). There was considerable variation in study design with regard to the type of right hemicolectomy performed (laparoscopic, open, limited or extended), timing of questionnaire administration and measurement tool used.

In 2017, Palmisano et al. [12] published a retrospective analysis of 225 patients who had laparoscopic or open right hemicolectomy, extended right hemicolectomy or ileocaecal resection between January 2005 and April 2014. Patients with metastatic disease or having surgery for palliation of their symptoms were excluded from the analysis.

Questionnaires were used to study patients’ perceptions of bowel activity and quality of life. Before surgery, patients completed two modules of the European Organization for Research and Treatment of Cancer (EORTC) questionnaire [13] along with the Gastrointestinal Quality of Life Index (GIQLI) questionnaire [14]. The GIQLI questionnaire was also administered 2 to 6 weeks after surgery, with all three questionnaires being again administered 3 to 6 months after surgery.

The study showed a trend towards improvement of bowel function after surgery. The majority of the patients interviewed reported satisfactory bowel function after right hemicolectomy. A significant correlation between diarrhoea and extended right hemicolectomy at 3 months was found; however, at 6 months, the association diminished. No definition of extended right hemicolectomy was given. The authors hypothesised that diarrhoea in the early postoperative period is caused by the loss of the terminal ileum, causing diminished bile salt absorption leading to an increased laxative effect, along with the loss of the ascending and transverse colon, thereby reducing the reabsorption of electrolytes and water from bowel content. They did not confirm this hypothesis with any diagnostic tests. They also described animal models that have shown that there is a compensatory adaption by the intestine to a loss of mucosal surface area [15, 16], which they suggested may explain the improvement in bowel function in the months after surgery. The study was limited as the results were based on subjective patient responses and patients with cancer ‘cured’ of disease may be more likely to report positive outcomes than those treated for benign disease.

A 2013 study by Theodoropoulos et al. [17] aimed to prospectively determine the health-related changes in the quality of life of 85 patients who had elective laparoscopic colectomy for colorectal cancer [17] in which participants were given questionnaires to assess quality life at 1, 3, 6 and 12 months postoperatively. Of the 85 patients in the study, 22 (25.8%) had laparoscopic right colectomy. All of the patients were treated for colorectal cancer at the same hospital. Along with EORTC and GIQLI questionnaires, a Short Form Health Survey questionnaire (SF-36) [18] was completed. Those with left-sided resection reported significantly worse continence and greater impact on activities of daily living due to emotional problems at 1 month postoperatively and worse social functioning and greater embarrassment at 6 months, when compared with those who had right-sided resections. However, it was thought that this may be contributed to the presence of a stoma in some respondents, which was less likely after right-sided resections. Twelve months after surgery, there were no differences noted in the outcomes between patients who had right-sided resection compared to left-sided or rectal resection. Whilst a subgroup analysis of right-sided resections was performed, this only included 22 patients and the focus was on the comparison between this and other resections. Across all types of resection including right-sided, there was an overall improvement in health-related quality of life at 12 months following surgery with a significant reduction in bowel symptoms. The authors concluded that the health-related quality of life improved after surgery, with a particular improvement in emotional status. This is in general agreement with the study by Palmisano [12].

Another study by the same author in 2013 investigated gastrointestinal function after treatment for colorectal cancer in a larger cohort of 289 patients, of which 79 (27.3%) had right-sided resection [19]. The patients were recruited from the same site as their earlier study and as such some patients may have been included in both studies. Both studies were included in the review due to the larger sample size in the later study and the differences in questionnaire use and timing. The cohort was evaluated preoperatively at 3, 6 and 12 months after surgery. Patients who had right-sided resections reported less excessive gas, constipation and uncontrolled stools at 3 months compared with left-sided, but this difference disappeared after 3 months. The use of repeated questionnaires at multiple time points allowed for temporal comparison of outcomes as patients adapt postoperatively; however, there was a very small sample size. This study concurred with Palmisano et al. [12] that in general, bowel function following all types of colectomy is satisfactory and that any dysfunction normalises over time.

Magdeburg et al. [20] investigated the functional outcome following colonic resection and the subsequent impact on the quality of life in a cohort of 297 patients between 2005 and 2013, with patients who had right hemicolectomy accounting for 28.6% of the cohort. Resections for both cancer and diverticular disease were included in the analysis. Two questionnaires were used in the study: the Short Form 12 (SF-12) [21] was used to measure the general quality of life, and the Faecal Incontinence Quality of Life scale (FIQL) [22] was used to measure the quality of life specifically related to bowel function. Following right-sided resection, significantly more patients reported liquid stool more than once per month (45.3% vs 38.7% *p* = 0.011). However, there was no overall difference in the quality of life between the groups using either score.

A study by Brigic et al. [23] compared bowel function in two cohorts against a control group. Ninety-one preoperative patients were included in the study (classified as the ‘early’ group) and compared with 85 patients who had surgery 2 to 4 years previously (used to assess intermediate bowel function) and 85 healthy relatives used as controls. All types of resection for cancer more than 15 cm above the anal verge were included for analysis. Patients who had low anterior resection were excluded. The study used the Memorial Sloan-Kettering Cancer Centre (MSKCC) bowel function questionnaire that was designed to assess bowel function after rectal resection [24, 25]. The MSKCC questionnaire does not measure the quality of life and had previously been validated against the FIQL and EORTC questionnaires [24].

The study concluded that if the frequency of bowel movements is considered alone, one-third of patients at 6 and 12 months and one-quarter of patients at 2 and 4 years after bowel resection have a significant increase in frequency compared with control. However, patients did not perceive this as a problem. There was no difference in the quality of life between right- and left-sided resections. However, 2 to 4 years after right-sided resection, patients reported a worse frequency score on the MSKCC questionnaire. The frequency component includes questions regarding number of bowel movements per 24 h, stool consistency and ability to get to the toilet on time. The authors also mention the potential limitations of selection bias and recall bias in their study.

The secondary aim was to identify any treatment modalities that exist for treating bowel dysfunction after right hemicolectomy. The established treatments for diarrhoea following right hemicolectomy focus on treating the underlying cause and expert clinical review is often beneficial [26]. Bile acid sequestrants such as cholestyramine and colesevelam are the first-line treatment when bile acid malabsorption is proven or suspected [27]. However, there are no studies that investigate the use of bile acid sequestrants after operative treatment of colon cancer. Small bowel bacterial overgrowth can be treated with antibiotic therapy, although there is a lack of consensus on dose and duration [28]. A systematic review found that rifaximin appears to be safe and effective for the treatment of small bowel bacterial overgrowth [29]; however, further high-quality studies are required. The use of probiotics for small bowel bacterial overgrowth is not proven and has only been assessed in pilot studies [30, 31]. The use of anti-diarrhoeal agents to control symptoms following intestinal resection is sparsely studied in cancer patients. Two small studies found that loperamide improved stool consistency and reduced the number of stools in patients following resection or with ileocolic disease [32, 33].

Only two studies investigated the effect of an intervention on postoperative bowel function. One focused on different types of anastomosis formation [34], whilst another investigated the effect of probiotic administration [35].

The ISOVANTI trial [34] was a double-blind randomised trial comparing isoperistaltic with antiperistaltic ileocolic anastomoses in patients having laparoscopic right hemicolectomy for cancer. A total of 108 patients were included, with 54 randomised to each arm. The primary aim of the study was to compare the safety and feasibility of the two techniques, and the secondary endpoint was to assess long-term functional outcomes and quality of life at 12 months. Patients were interviewed and GIQLI Questionnaires were administered at 1, 6 and 12 months postoperatively. Ten out of 51 (18.5%) patients in the isoperistaltic group reported diarrhoea (> 3 stools/day) compared with 16 out of 52 (29.6%) in the antiperistaltic group; this however did not reach statistical significance. After 1 year, the rate of chronic diarrhoea (defined as more than three liquid stools per day for a period of longer than 4 weeks) was 24% in the isoperistaltic group and 31.4% in the antiperistaltic group. The authors concluded that regardless of anastomosis type, patients demonstrated significant improvement in the quality of life postoperatively compared with their preoperative score (*p* < 0.001). Whilst chronic diarrhoea was noted, it was not correlated with a worse quality of life, which agrees with previous work [23].

Ohigashi et al. [35] investigated whether probiotics were effective in improving bowel function after colorectal resection. The authors conducted a questionnaire-based study to assess 193 patients and assess their response to probiotic treatment. SF-36, EORTC QLQ-C30 and the Wexner Incontinence Score [36] were the questionnaires used. Following right-sided colonic resection, stools were looser and the night time defaecation frequency was higher than in the left-sided resection group. Role function and physical function as assessed by the quality of life questionnaires were lower in the right resection group compared with left, indicating a worse outcome.

A probiotic containing *Bacillus natto* and *Lactobacillus acidophilus* was administered to 18 patients after right-sided resection. All patients who completed the initial questionnaire were asked to take probiotics. The average time from surgery to taking probiotics was 932 days. Three months after probiotic treatment, questionnaires assessing bowel function and quality of life were completed. Defaecation frequency and feeling of incomplete evacuation were significantly improved after probiotic treatment at 3 months. Global quality of life was also significantly improved. The administration of probiotics showed improvements in stool frequency and softness which it was suggested may be due to changes in intestinal flora. The study did not compare the pre- and post-probiotic microbiome of the colon. Whilst the authors report some significant findings regarding the efficacy of probiotics, there were only a small number of patients and there was no placebo group for comparison. The study concluded that bowel dysfunction following right hemicolectomy can persist for more than 2 years and did not concur with other studies suggesting that changes in bowel function were temporary and improved with time.

Two studies [37, 38] compared the effect of a more radical lymph node dissection to that of a conventional right hemicolectomy. Extended dissection to include the lymph nodes that surround the superior mesenteric vessels has been shown in some studies to convey a survival benefit [39, 40]; however, this is not widely performed in Western Europe. Thorsen et al. [37] investigated the impact of right colectomy with D3 extended mesenterectomy compared to right colectomy alone. Bowel frequency and urgency were increased in both groups. The only significant difference between the groups was that bowel frequency was greater in the mesenterectomy group; however, this did not affect gastrointestinal quality of life. Forty-nine percent (24/49) of patients who had standard right colectomy stated that their bowel habits did not bother them and all of the standard right colectomy patients reported less than or equal to three bowel movements per day. The median time to postoperative interview to assess bowel function was significantly different between the groups, 14.9 months in the mesenterectomy compared with 34.4 months in the controls. The study concludes that small bowel denervation may contribute less towards bowel dysfunction than previously thought.

Bertleson et al. [38] conducted a retrospective study including a total of 623 patients investigating long-term bowel dysfunction in patients who had a conventional right colectomy compared to right-sided complete mesocolic excision. The primary outcome measures were diarrhoea, four or more bowel movements per day and impact on the quality of life. The Bristol Stool Scale [41] measured diarrhoea rate and the EORTC QLQ-C30 was used to assess the quality of life. The median time after surgery that the questionnaire was administered was 4 years. Thirteen percent (40/307) patients reported diarrhoea in the conventional right colectomy group and 21.5% (68/316) reported bowel function impacted on their quality of life. This study had the largest sample size of patients having right hemicolectomy in this review. No comment can be made about preoperative bowel function in the study groups. This study made a clear anatomical definition between right hemicolectomy and extended right colectomy for inclusion criteria. Whilst there were no significant differences in bowel function between conventional right colectomy and complete mesocolic excision, the study found that bowel function had a moderate to severe impact of quality of life in 20% of patients having right-sided resection.

## Discussion

This systematic review has evaluated the effect of right hemicolectomy on bowel function. Loose stool, increased bowel frequency and/or nocturnal defaecation following right-sided colectomy occurs in approximately one in five patients. Some of these symptoms may improve spontaneously with time. How much this affects the quality of life varies between studies. The fact that some studies report no or little difference in outcome between right-sided and rectal resections actually suggests poor function after right-sided resection.

These findings are consistent with our understanding of the functional role of the ileocaecal valve, terminal ileum and right colon. A 1978 study found that the site of resection was the most important prognostic factor predicting intestinal malabsorption following extensive small bowel resection [42]; in particular the removal of the ileocaecal valve and right colon resulted in prolonged malabsorption. The mechanism for this may be impaired absorption of bile salts, resulting in more bile acids entering the colon and causing diarrhoea [43, 44] due to increased water secretion and colonic motility [45–47]. The prevalence rate of bile acid malabsorption following ileal resection or right hemicolectomy has been reported as being between 89 and 91% [48] [49] and may occur to a severe degree after as little as 10 cm of terminal ileum is resected. Secondly, the right colon is the main site of water reabsorption; therefore, the loss of this capacity may also contribute to looser stools [50]. Thirdly, the ileocaecal valve plays an important barrier function in preventing entry of colonic bacteria into the small bowel, and its removal can promote the development of small bowel bacterial overgrowth [51].

Some studies [12, 19, 52] reported improvement in bowel function after 12 months, which may be secondary to the physical adaption of the remaining bowel or patients learning to manage their symptoms. The process of structural adaptation following bowel surgery is not well understood, and the majority of studies are based on animal subjects and preclinical data. A review by Tappenden [53] summarised the different mechanisms that contribute to postoperative bowel adaptation including structural mucosal changes, angiogenesis, enterocyte differentiation and slowed intestinal transit. A study in adults post jejuno-ileal bypass shows that hypertrophy of villi leads to an increase in the absorptive capacity of the remaining bowel [54]. Studies investigating functional bowel adaptation after resection in adult humans are sparse and of a small sample size. Many factors can influence how patients respond to the quality of life questionnaires including those with progressive disease may be likely to report worse outcomes than those cured. Some reported ‘improvement’ of bowel function has been shown to occur because these symptoms become part of a patient’s everyday life; their ‘normality’ is adjusted and symptoms are tolerated even when severely limiting activities [55].

The limitations of this review relate to the methodology of the included studies. In general, sample sizes were modest and all studies relied on subjective measures of bowel function. None of the studies used objective postoperative testing to investigate the possible causes of diarrhoea. None of the studies reported the use of constipating drugs which may result in improved symptoms. As all of the studies were questionnaire based, they were subject to recall bias. The questionnaires used were very variable across studies, and some focused on quality of life and others bowel function. This may in part be due to the lack of a validated tool to assess bowel function specifically after segmental colonic resection. Clinical follow-up also varied between studies, with only three studies assessing bowel function past 12 months. Some studies had a wide range in questionnaire response time, which makes interpretation of results difficult if not impossible. Early in the postoperative period, some patients are still recovering from the physical effects of major surgery, and this may negatively impact on subjective quality of life scores. The generally accepted view is that bowel adaptation following surgery occurs in the first 2 years in adults [56]. This suggests that studies with longer follow-up periods are required. Due to the mentioned limitations, it is not possible to draw firm conclusions about the impact of right hemicolectomy on bowel function; to do this, further studies with objective clinical outcomes as opposed to questionnaires would be required.

It was not possible to perform a meta-analysis due to heterogeneity of studies, in particular differences in the measurement tools used. Whilst right hemicolectomy is a well-established procedure, there can be many variations in approach (open, laparoscopic, robotic) and anastomosis technique (stapled, handsewn, intra/extra corporal anastomosis). In the long term, it seems unlikely that the surgical approach will have a long-term impact on bowel function. However, the type of anastomosis may affect gastrointestinal motility, and only one study in this review investigated this. The technical details of how the right hemicolectomy was performed were lacking across studies, with most articles not clearly defining ‘right hemicolectomy’. Furthermore, some studies included extended right hemicolectomy and ileocaecal resections.

## Conclusion

Right hemicolectomy can result in changes to bowel function. Hemicolectomy patients should be counselled about this preoperatively, and follow-up should be designed to identify significantly altered bowel function, and patients should be offered appropriate investigations and treatment for this. However, many patients seem to tolerate their change in bowel habit as a new normal. Further studies to assess preoperative and postoperative interventions that reduce the risk of bowel dysfunction following right hemicolectomy are warranted.
